# ANGPTL4 Attenuates Ang II-Induced Atrial Fibrillation and Fibrosis in Mice via PPAR Pathway

**DOI:** 10.1155/2021/9935310

**Published:** 2021-08-09

**Authors:** Xi Zhu, Xiaogang Zhang, Xinpeng Cong, Luoning Zhu, Zhongping Ning

**Affiliations:** Department of Cardiology, Shanghai University of Medicine & Health Sciences Affiliated Zhoupu Hospital, Shanghai 201318, China

## Abstract

Atrial fibrillation (AF) is the more significant portion of arrhythmia in clinical practice, with inflammation and fibrosis as its central pathological mechanisms. This study aimed to investigate angiopoietin-like 4 (ANGPTL4) effects on angiotensin II- (Ang II-) induced AF and its related pathophysiological mechanisms. C57BL/6J mice were randomized and divided into three groups: the control group, the Ang II group, and the ANGPTL4 group (Ang II with ANGPTL4 treatment). Mice were infused with Ang II (2000 ng/kg/min) and were administrated with recombinant human ANGPTL4 (rhANGPTL4, 20 *μ*g/kg/day) for 3 weeks. The fibrosis was evaluated with Masson's trichrome staining in the atrial myocardium. mRNA levels of IL-1*β*, IL-6, collagen I, and collagen III were measured using real-time qRT-PCR. Protein levels of PPAR*α*, PPAR*γ*, CPT-1, and SIRT3 were measured using Western blotting. Compared to the control group, the mice infused with Ang II showed electrocardiogram characteristics of AF, and this effect was markedly attenuated in ANGPTL4-treated mice. ANGPTL4 also reversed the increase in cardiomyocyte apoptosis, inflammation, interstitial collagen fraction, and collagen gene expression in mice with Ang II. Mechanistically, ANGPTL4 inhibited the activation of several fatty acid metabolism-related proteins, including PPAR*α*, PPAR*γ*, and CPT-1, and the expression of SIRT3 protein in atrial tissues. In conclusion, ANGPTL4 attenuates Ang II-induced AF and atrial fibrosis by modulation in the SIRT3, PPAR*α*, and PPAR*γ* signaling pathways.

## 1. Introduction

Atrial fibrillation (AF) is a persistent arrhythmia that originates from the abnormal atrial matrix and has a variety of inducing factors, such as heart failure, diabetes, and hypertension [1]. The pathogenesis of atrial fibrillation is complex. Atrial fibrosis is an important pathological feature of atrial structure in AF and is closely associated with its occurrence and maintenance [2]. Increased atrial fibrosis can be observed in the extracellular matrix (ECM) of atrial tissue of animal models and case biopsies of heart failure [3, 4]. The main manifestation of atrial fibrosis is ECM remodeling. The ECM not only provides stable support for myocardial cells and maintains the stability of myocardial cell structure but also plays an important role in the signal transduction between myocardial cells [5]. Therefore, atrial fibrosis is a key pathological process of AF, and reducing atrial fibrosis can effectively suppress the susceptibility to AF in animal models [6]. At present, the therapeutic strategies that prevent atrial fibrosis are urgently needed in AF, but its specific mechanism is still unclear.

Angiopoietin-like 4 (ANGPTL4) is a secretory protein regulated by PPAR*γ* under fasting conditions to affect lipid metabolism. Recent studies have shown that ANGPTL4 can regulate tumorigenesis, angiogenesis, vascular permeability, lipid metabolism, glucose, energy homeostasis, cell differentiation, wound healing, inflammatory response, and redox reaction. Physiological conditions such as fasting, hypoxia, pregnancy, lactation, and adipocyte differentiation lead to upregulation of ANGPTL4 expression. In addition, chronic caloric restriction, short-term hypothermia, a deficient calorie diet, a high-fat high-energy diet, and free fatty acids have been shown to increase plasma ANGPTL4 concentration. ANGPTL4 can play a vital role in lipid metabolism by inhibiting the activity of lipoprotein lipase (LPL), which is responsible for the hydrolysis of plasma triglyceride (TG) into free fatty acids [7]. Early studies showed that overexpression of ANGPTL4 decreases LPL activity and increases circulating triglyceride levels.

Conversely, ANGPTL4-deficient mice exhibit increased plasma LPL activity and decreased plasma triglyceride levels. Therefore, antibodies against ANGPTL4 showed reduced circulating TAG in mice [8]. It is worth noting that patients with AF had significantly higher levels of triglycerides [9], which indicate that the lowering effect of ANGPTL4 on plasma triglycerides might help reduce the risk of AF. Several studies have suggested that ANGPTL4 is associated with increased risk factors for cardiovascular disease and might act as a potential therapeutic target against cardiovascular diseases. For instance, plasma ANGPTL4 levels have been used to predict the risk of cardiovascular events [7]. ANGPTL4 also has shown protective effects on the cardiovascular injury. Triglyceride is hydrolyzed into free fatty acids to provide energy for the myocardium. Excessive free fatty acids can induce cardiomyocyte death. Free fatty acids can inhibit LPL and protect the myocardium by activating PPAR *β*/*δ* and inducing ANGPTL4 expression [10]. Simultaneously, overexpression of ANGPTL4 can improve glucose tolerance in high-fat-diet-induced obese mice, suggesting that ANGPTL4 may enhance insulin sensitivity. Insulin resistance is associated with a high risk of new-onset atrial fibrillation. However, the role of ANGPTL4 in AF and related mechanisms remains mostly unknown.

This study tested the hypothesis that ANGPTL4 can prevent atrial remodeling and fibrosis in angiotensin II- (Ang II-) induced mice model, potentially through modulation of the fatty acid metabolism signaling pathway.

## 2. Materials and Methods

### 2.1. Animals and Treatment

Male C57BL/6 mice (8 weeks old) were infused with saline or Ang II (2000 ng/kg/min) using osmotic minipumps for 3 weeks. The recombinant human ANGPTL4 (rhANGPTL4, 20 *μ*g/kg) (HY-P7507, MCE, Burlington, NJ, USA) saline was injected intraperitoneally once a day for 3 weeks. This study was approved by the Animal Care and Use Committee of Shanghai Pudong Zhoupu Hospital. The guide conducted all experimental procedures for the Care and Use of Laboratory Animals (NIH publication no. 85-23, revised 1996).

### 2.2. Arrhythmia Inducibility and Duration

After 3 weeks of ANGPTL4 administration, the mice were anesthetized by intraperitoneal injection of 1% pentobarbital sodium, and eight-electrode catheters were inserted via the jugular vein to the right atrium to record electrocardiograms. At the end of the Ang II infusion, the esophageal pacemaker electrode was inserted into the esophagus near the atrium to enable it to capture the atrial electrocardiogram. Then, the output end of the pacemaker electrode was connected with the psychopharmacology electronic stimulator. The stimulator parameters were set (voltage: 20 V; current: 4 mA; wave width: 6 ms; interval: 20 ms). Then, the intracardiac pacing was performed in mice. The inducibility of AF was measured by stimulating the mice for 10 s at an interval of 5 min. The next stimulation was performed after the mice recovered sinus rhythm. When the typical atrial fibrillation wave appeared in the ECG of mice, the F wave accompanied by the P wave disappeared. The RR interval appeared irregular; it could be considered atrial fibrillation. The RR interval changes, QRS interval, and QT interval were observed and analyzed before and after intracardiac pacing.

### 2.3. Histopathologic Examinations and Immunohistochemistry

More than 3 weeks after the ANGPTL4 administration, the mice were anesthetized with pentobarbital sodium, decapitated, and the atrial tissue was fixed with 4% paraformaldehyde for 4 hours, paraffin-embedded, and cut serially into section (thickness of 4 *μ*m). Tissues were mounted on positive-charged slides and were deparaffinized in xylene and rehydrated. Slides were stained with hematoxylin-eosin, Masson's trichrome, or incubated with polyclonal anti-mice rabbit anti-*α*-SMA antibody (ab5694; Abcam, Bristol, UK) at 37°C for 20 minutes, followed by biotinylated HRP conjugated secondary antibody. The slides were washed and counterstained. Images were photographed from ten random fields per sample (×200 magnification) and were analyzed by ImageJ.

### 2.4. Real-Time Quantitative PCR (RT-qPCR)

Total RNA was extracted from mice atrial tissues using TRizol (Invitrogen, USA) and was reversely transcribed into complementary DNA (cDNA). The mRNA was amplified by RT-qPCR using SYBR Green reagent (TaKaRa, Japan) in an ABI Prism 7700 Real-Time PCR system (Applied Biosystems, USA). Primer sequences were as follows: ANGPTL4 (forward: 5′-GAG GTC CTT CAC AGC CTG CA-3′; reverse: 5′- TGG GCC ACC TTG TGG AAG AG-3′); IL-1*β* (forward: 5′-GCA ACT GTT CCT GAA CTC AAC T-3′; reverse: 5′-ATC TTT TGG GGT CCG TCA ACT-3′); IL-6 (forward: 5′-GTT TTC TGC AAG TGC ATC ATC G-3′; IL-6 reverse: 5′-GGT TTC TGC AAG TGC ATC ATC G-3′); collagen I (forward: 5′-GGA CAC TAC TGG ATC GAC CTA AC-3′; reverse: 5′-CTC ACC TGT CTC CAT GTT GCA-3′); collagen III (forward: 5′-CTA CCT TGC TCA GTC CTA TGA GTC TAG A-3′; reverse: 5′-TCC CGA GTC GCA GAC ACA TAT-3′); and GAPDH (forward: 5′-ACT CCA CTC ACG GCA AAT TC-3′; reverse: 5′- TCT CCA TGG TGG TGA AGA CA-3′). Experiments were performed in triplicate. The 2−*ΔΔ*Ct method was used to calculate mRNA expression. GAPDH mRNA was used as an internal control.

### 2.5. Western Blotting

The mice's left atrium was homogenized in RIPA lysis buffer (P0013 B, Beyotime Biotechnology, China) containing protease inhibitor cocktail (1 : 100, Sigma). Total protein (50 *μ*g) was separated by 10% SDS-PAGE gels and then separated protein was transferred onto a PVDF membrane. The membrane was blocked with 5% skimmed milk and subjected to primary antibodies overnight at 4°C against ANGPTL4 (1 : 200; ab196746, Abcam), PPAR*α* (1 : 200; sc-398394, Santa Cruz), PPAR*γ* (1 : 200; sc-7273, Santa Cruz); CPT-1 (1 : 500; ab234111, Abcam), SIRT3 (1 : 200; ab246522, Abcam), and *ß*-actin (1 : 1000; ab8226, Abcam), followed by incubation with HRP-conjugated secondary antibody. Protein bands were visualized using Enhanced Chemiluminescence Plus (Millipore, MA, USA). Relative expression of proteins was normalized to *ß*-actin.

### 2.6. Statistical Analysis

Data were presented as the mean ± standard deviation (SD) from at least three independent experiments. All statistical analysis was carried out using the SPSS software (version 20.0, SPSS, Inc., USA). The differences between the groups were tested using one-way ANOVA. *P* < 0.05 was the criteria of statistical significance.

## 3. Results

### 3.1. ANGPTL4 Attenuated Ang II-Induced AF

**Figure 1:**
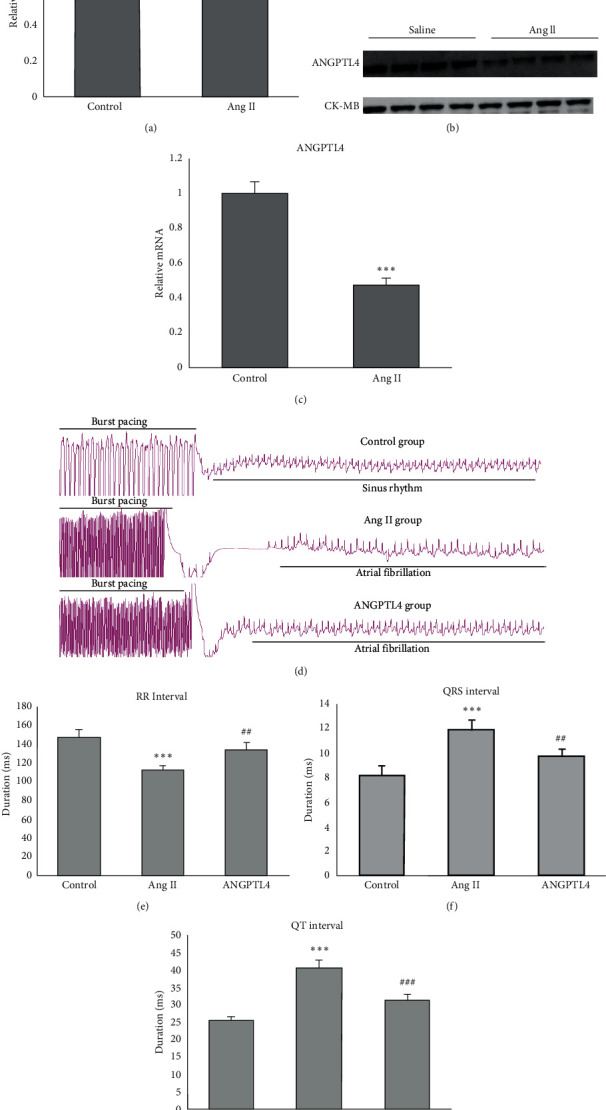
ANGPTL4 attenuates Ang II-induced AF. Mice were infused with Ang II and treated with ANGPTL4 (20 *μ*g/kg, once a day) for 3 weeks. (a) ANGPTL4 mRNA levels are significantly elevated in atria of Ang-II-induced mice models. (b) Representative bands of the ANGPTL4 protein level in Ang-II-induced mice models. (c) Quantification of ANGPTL4 protein level in Ang-II-induced mice models. (d) Representative atrial electrogram recordings after burst pacing in mice. (e) RR interval, (f) QRS interval, and (g) QT interval after burst pacing in mice of three groups are shown (*n* = 6 mice per group). ^*∗*^^*∗*^^*∗*^*P* < 0.001 vs. control group; ^##^*P* < 0.01, ^###^*P* < 0.001 vs. Ang II group.

To investigate the expression of ANGPTL4 in AF, we measured the ANGPTL4 levels in saline-infused and Ang-II-infused mice. Compared to the saline-infused mice, Ang-II-infused mice showed significantly upregulated ANGPTL4 mRNA and protein expression in the left atrial (Figure 1(a)–1(c)). To investigate the role of ANGPTL4 in regulating AF development, the electrocardiogram was recorded in mice infused with Ang II with or without recombinant human ANGPTL4 (20 *μ*g/kg/day). Each mouse was given 10 times electrical stimulation, with 200 times in each group. The number of successful AF episodes was 153 times in the Ang II group and 68 times in the ANGPTL4 group. So, ANGPTL4 reduced the Ang II-triggered inducibility of AF in mice (Ang II group: 76.5%; ANGPTL4 group: 34.0%). There was no atrial fibrillation in the control group. After transesophageal rapid atrial pacing, the mice in the AF group showed typical atrial fibrillation attack, and a P wave replaced the P wave with different RR intervals. While the control group showed sinus rhythm, including P wave and the same RR intervals (Figure 1(d)). After rapid esophageal atrial pacing, the heart rate was significantly declined in the ANGPTL4 group compared to the Ang II group, with a higher RR interval (Figure 1(e)) and lower QRS interval (Figure 1(f)) and QT interval (Figure 1(g)).

### 3.2. ANGPTL4 Attenuated Ang II-Induced Inflammation and Apoptosis

**Figure 2:**
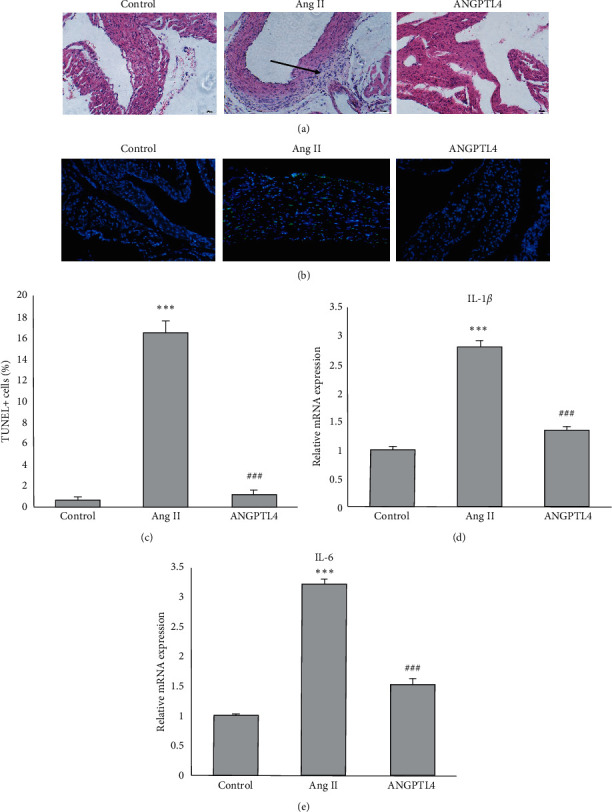
ANGPTL4 inhibits Ang II-induced atrial inflammation and apoptosis. (a) Representative image of HE staining in the atrial tissues of mice. The arrow indicates the infiltrated inflammatory cells. (b) TUNEL staining of atrial tissues for apoptosis detection. (c) The quantification of TUNEL positive cells in atrial tissues (*n* = 6 mice per group). RT-qPCR analyses of (d) IL-1*β* and (e) IL-6 levels in atrial tissues. *n* = 6 mice per group, *n* represents the number of animals. ^*∗*^^*∗*^^*∗*^*P* < 0.001 vs. control group; ^###^*P* < 0.001 vs. Ang II group.

Histological pathology was performed using HE staining to assess the influence of ANGPTL4 on inflammation and apoptosis of atrial tissue. ANGPTL4 attenuated the Ang II infusion which induced increase in inflammatory cell infiltration in atrial tissue of mice (Figure 2(a)). Moreover, TUNEL staining revealed that the Ang II-induced atrial apoptosis in WT mice decreased in ANGPTL4-treated mice (Figure 2(b)). Quantification analysis showed that the TUNEL positive cells were significantly lower in the ANGPTL4 group than the Ang II group (Figure 2(c)). Accordingly, the mRNA levels of two proinflammatory cytokines, IL-1*β* and IL-6, significantly lowered the ANGPTL4 group than in the Ang II group (Figures 2(d) and 2(e)).

### 3.3. ANGPTL4 Attenuated Ang II-Induced Atrial Fibrosis

**Figure 3:**
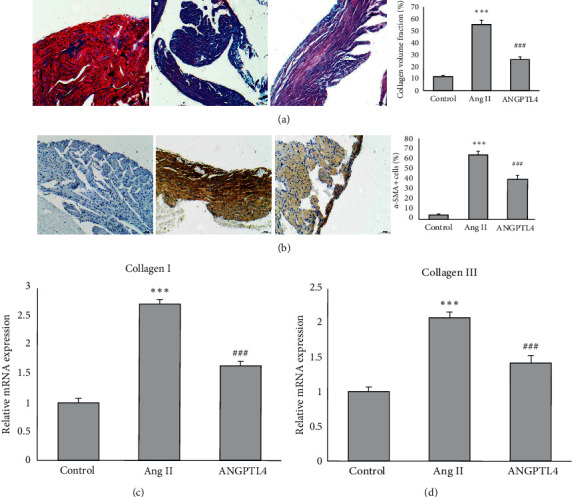
ANGPTL4 suppresses Ang II-induced atrial fibrosis. (a) Representative images of atrial fibrosis, which was stained with Masson trichrome. The fibrotic area was quantified. (b) Immunohistochemistry image of *a*-SMA. The percentage of *a*-SMA-positive cells is quantified. RT-qPCR analyses show the mRNA levels of collagen I and collagen III in atrial tissues. *n* = 6 mice per group, *n* represents the number of animals. ^*∗*^^*∗*^^*∗*^*P* < 0.001 vs. control group; ^###^*P* < 0.001 vs. Ang II group.

Ang II treatment significantly increased the atrial fibrotic area, while this effect was remarkably attenuated by ANGPTL4 (Figure 3(a)). Moreover, immunohistochemistry showed the ANGPTL4 markedly attenuated Ang II-induced increase in the number of *a*-SMA-positive myofibroblasts in atrial tissue (Figure 3(b)). Ang II increased mRNA expression of collagen I and III in atrial tissue of mice, but the expression of these two fibrotic markers were markedly reduced by ANGPTL4 treatment (Figures 3(c) and 3(d)).

### 3.4. ANGPTL4 Modulated the Expressions of Proteins Associated with Fatty Acid Metabolism

**Figure 4:**
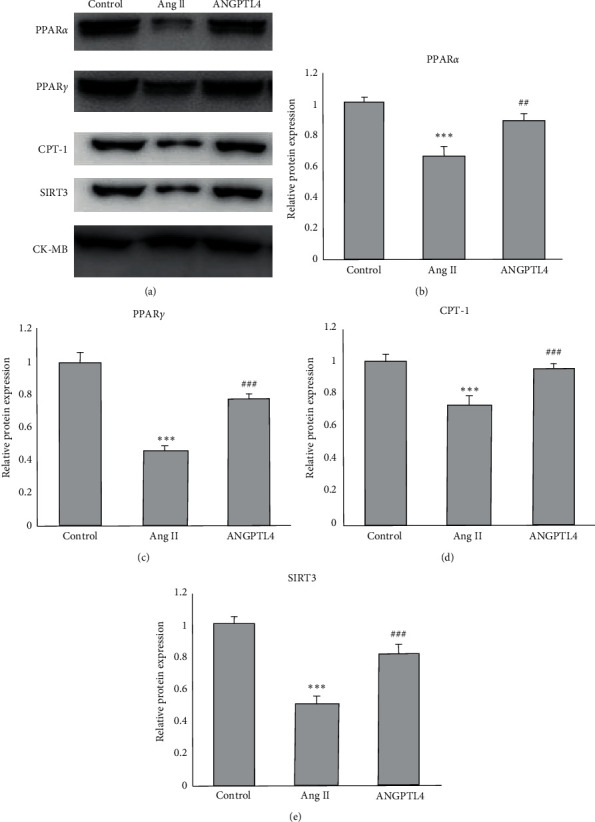
Treatment with ANGPTL4 regulates protein levels of fatty acid metabolism. (a) Representative bands of Western blot was performed for (b) PPAR*α*, (c) PPAR*γ*, (d) CPT-1 and (e) SIRT3 (*n* = 5). ^*∗*^^*∗*^^*∗*^*P* < 0.001 vs. control group; ^##^*P* < 0.01, ^###^*P* < 0.001 vs. Ang II group.

Western blot was performed to measure protein level of PPAR*α*, PPAR*γ*, CPT-1, and SIRT3 (Figure 4(a)). Ang II infusion decreased the protein expression in PPAR*α*, PPAR*γ*, CPT-1, and SIRT3, and this indicated that impaired fatty acid metabolism is involved in the process of atrial fibrosis. Compared with the mice with Ang II infusion alone, mice with Ang II and ANGPTL4 cotreatment showed significantly lower levels of these proteins in atrial tissue (Figures 4(b)–4(d)).

## 4. Discussion

This study firstly showed the protective effect of ANGPTL4 treatment in Ang II-infused atrial tissues. ANGPTL4 mRNA and protein expression were reduced in the atrial tissue of Ang-II-infused mice. Ang II increased AF susceptibility and atrial fibrosis, and ANGPTL4 significantly attenuated Ang II-induced increase in arrhythmia, fibrosis, inflammation, and apoptosis in atrial tissue. The potential mechanism may be associated with the inhibition of multiple signaling proteins, such as PPAR*α*, PPAR*γ*, CPT-1, and SIRT3. Taken together, we demonstrated for the first time that ANGPTL4 is a potential protective molecule in Ang II-induced AF.

The study speculates the hypothesis that ANGPTL4 is a protective factor of atrial fibrillation, as evidenced by reduced expression of ANGPTL4 in Ang-II-infused mice and suppressive effect of ANGPTL4 on abnormal atrial electrogram changes in mice with Ang II infusion. These results are in accordance with our previous study that significantly lowers serum ANGPTL4 which were observed in AF patients and associated with cardiac hypertrophy, oxidative stress, and inflammation [11]. Abnormal fatty acid metabolism is an essential promoting factor of atrial fibrillation. The expression of fatty acid-binding protein 3 (FABP3), a gene encoding fatty acid transport, was decreased in atrial tissue of AF patients [12]. Also, AF patients showed increased saturated fatty acid levels. They decreased polyunsaturated fatty acid levels, which may be related to the enhancement of inflammation, suggesting that free fatty acid levels may play a vital role in the development and progress of AF [13]. A series of studies have confirmed that omega-3 polyunsaturated fatty acid (N-3 PUFA) has antiarrhythmic effects in AF, and the associated mechanisms include electrophysiological, anti-inflammatory, and antifibrosis effects [14]. ANGPTL4 can also be strongly induced by omega-3 long-chain fatty acids in human's subjects [15]. The results indicate ANGPTL4 might be a mediator of polyunsaturated fatty acid in antiarrhythmic effects, and our study confirms this hypothesis in an in vivo AF model induced by Ang II.

The present study shows that ANGPTL4 administration reversed Ang II-induced atrial inflammation, apoptosis, and fibrosis. Ang II is a contributing factor of AF and can stimulate atrial fibrosis and inflammation, thus increasing AF inducibility [16]. Inflammation is implicated in AF's pathophysiology, and excessive inflammatory mediators diffuse into atrial tissue altering its structural and electrical properties [17]. ANGPTL4 also shows a potent anti-inflammatory effect. ANGPTL4 treatment increased the antiinflammatory macrophages in the myocardial infarction animal model and markedly improved cardiac function [18]. Our results showed that the mRNA expression of two proinflammatory cytokines, including IL-1*β* and IL-6, was decreased in atrial tissues of mice with ANGPTL4 administration. Atrial fibrosis is also an essential pathophysiological contributor of AF and results in the replacement of dead cardiomyocytes with ECM tissue and fibroblasts, thus damaging electric conduction of atrial tissue [19]. Recent data have demonstrated that ANGPTL4 can suppress phenylephrine-induced cardiomyocyte hypertrophy, another characteristic of myocardial remodeling [20]. Here, our results showed that compared to mice with Ang II alone, mice with Ang II and ANGPTL4 showed significantly suppressed atrial fibrosis and increased expression of *a*-SMA, collagen I, and collagen III. Therefore, these results provide a new mechanism underlying the protective effect of ANGPTL4 in AF. Our results also proved the previous report that ANGPTL4 inhibits scar-associated collagen production in fibroblasts through the *ß*-catenin-ID3 pathway [21]. Whether these pathways involve the suppressive effect of ANGPTL4 in atrial fibrosis is unknown and deserves further investigation [22].

Our study shows that the expressions of proteins associated with fatty acid metabolism, such as PPAR*α*, PPAR*γ* and CPT-1, were higher in mice with ANGPTL4. In general, fatty acid is the primary typical energy source for cardiac function. In contrast, under pathological conditions, the myocardium converts energy sources from fatty acid to glucose, thus reducing energy supply [23]. PPAR*α* is a primary transcriptional regulator of fatty acid oxidation, while PPAR*γ* can regulate the expression of genes with lipid metabolism [24]. PPAR*α* involves regulating myocardial metabolism and is related to attenuation of AF through regulation of lipid metabolism in the heart [25, 26].

Furthermore, lower serum PPAR*γ* was observed in elderly AF patients [27], and this indicates it is a potential target of AF. PPAR*γ* activator could reduce AF susceptibility by promoting atrial cell survival [28] and inhibit cardiac fibrosis caused by several risk factors of AF, such as diabetes, hypertension, myocardial infarction, and heart failure [29]. PPAR*α* and PPAR*γ* are also the activators of transcription and expression of the ANGPTL4 gene [30]. The PPAR-*α*/ANGPTL4 pathway mediates endothelial barriers in high glucose-induced cardiac microvascular endothelial cells [31]. However, it seems there is mutual regulation between PPAR*α* and ANGPTL4, as ANGPTL4 also regulates PPAR*α* [20]. This evidence indicates that ANGPTL4 might mitigate atrial fibrosis via the regulation of PPAR*α* and its downstream target CPT-1. Our results also showed that ANGPTL4 suppressed the expressions of SIRT3 proteins. SIRT3 was involved the inhibition of Ang-II-induced cardiac fibrosis through the ROS-TGF-*β*1 pathway [32]. Furthermore, SIRT3 inhibited myocardial fibrosis and apoptosis after myocardial infarction by regulating the *ß*-catenin/PPAR*γ* signal pathway [33]. This can explain why administration of ANGPTL4 also downregulates the expression of PPAR*γ* in atrial tissue of AF mice.

There are some limitations of this study. Firstly, the expression of ANGPTL4 in atrial tissue of AF mice is unknown. Whether Ang II modulates, ANGPTL4 requires further research, and the expression of ANGPTL4 should also be verified in a clinical sample, including serum and excised myocardial tissue of AF patients. Secondly, the changes in fatty acid metabolism in epicardial adipose tissue should be investigated, closely associated with atrial fibrosis and inflammation.

In conclusion, ANGPTL4 administration reduces susceptibility to AF by inhibiting atrial inflammation and fibrosis. The potential mechanism of ANGPTL4 may be associated with Ang II-induced changes in fatty acid metabolism. PPAR*α*, PPAR*γ* and SIRT3 are the major downstream pathway proteins of ANGPTL4 in atrial tissue of Ang II-induced mice. This study indicates that ANGPTL4 has potential clinical value for AF associated with hypertension and other disorders.

## Data Availability

The data used to support the findings of this study are available from the corresponding author upon request.
